# Universality of High-Strength Tensors

**DOI:** 10.1007/s10013-021-00522-7

**Published:** 2021-09-28

**Authors:** Arthur Bik, Alessandro Danelon, Jan Draisma, Rob H. Eggermont

**Affiliations:** 1 MPI for Mathematics in the Sciences, Leipzig, Germany; 2 Eindhoven University of Technology, Eindhoven, Netherlands; 3 University of Bern, Bern, Switzerland

**Keywords:** Strength, Polynomial functor, Infinite tensors, GL-varieties, 14R20, 15A21, 15A69, 20G05

## Abstract

A theorem due to Kazhdan and Ziegler implies that, by substituting linear forms for its variables, a homogeneous polynomial of sufficiently high strength specialises to any given polynomial of the same degree in a bounded number of variables. Using entirely different techniques, we extend this theorem to arbitrary polynomial functors. As a corollary of our work, we show that specialisation induces a quasi-order on elements in polynomial functors, and that among the elements with a dense orbit there are unique smallest and largest equivalence classes in this quasi-order.

## Introduction

Let *K* be an algebraically closed field of characteristic 0. For partitions *λ* of integers *d* ≥ 1, denoted as *λ* ⊩ *d*, we consider the corresponding Schur functors *S*_*λ*_. We refer the reader to [17] or [16, Lecture 6] for an introduction to these objects. For a tuple $\underline {\lambda }=[\lambda _{1},\ldots ,\lambda _{k}]$ of partitions *λ*_*i*_ ⊩ *d*_*i*_ ≥ 1, we denote $S_{\lambda _{1}}\oplus \cdots \oplus S_{\lambda _{k}}$ by $S_{\underline {\lambda }}$. For finite-dimensional vector spaces *V*, *W* and a linear map *φ* : *V* → *W*, we get a linear map

$$ S_{\underline{\lambda}}(\varphi)\colon~S_{\underline{\lambda}}(V)\to S_{\underline{\lambda}}(W) $$

that depends polynomially on *φ* and satisfies $S_{\underline {\lambda }}(\text {id}_{V})=\text {id}_{S_{\underline {\lambda }}(V)}$ and $S_{\underline {\lambda }}(\varphi \circ \psi )=S_{\underline {\lambda }}(\varphi )\circ S_{\underline {\lambda }}(\psi )$ whenever the former makes sense. In particular, taking *V* = *W* and restricting our attention to invertible *φ*, we find that $S_{\underline {\lambda }}(V)$ is a polynomial representation of the group GL(*V* ).

### *Example 1*

For *λ* = (*d*), *S*_*λ*_(*V* ) = *S*^*d*^*V*, the *d*-th symmetric power of *V*. If *x*_1_,…,*x*_*n*_ is a basis of *V*, *S*_(*d*)_(*V* ) is the space of homogeneous polynomials of degree *d* in *x*_1_,…,*x*_*n*_.

For two tuples $\underline {\lambda }$, $\underline {\nu }$ of partitions, we write $\underline {\nu } \lessdot \underline {\lambda }$ when the number of occurrences of every partition *μ* ⊩ *d* in $\underline {\nu }$ is at most the number of occurrences of *μ* in $\underline {\lambda }$, where *d* is the maximal integer for which these numbers differ for some *μ*.

### *Example 2*

We have $[(1),(1),(1,1),(3)] \lessdot [(2),(3),(2,1)]$.

Let $\underline {\lambda }$ be a tuple of partitions of positive integers. The following dichotomy is our first main result.

### **Main Theorem I 1**

Let $\mathcal {P}$ be a property that, for each finite-dimensional vector space *V*, can be satisfied by some elements of $S_{\underline {\lambda }}(V)$. Assume that $S_{\underline {\lambda }}(\varphi )(f)\in S_{\underline {\lambda }}(W)$ satisfies $\mathcal {P}$ for every element $f\in S_{\underline {\lambda }}(V)$ satisfying $\mathcal {P}$ and every linear map *φ*: *V* → *W*. Then either $\mathcal {P}$ is satisfied by all elements of $S_{\underline {\lambda }}(V)$ for all *V* or else all elements satisfying $\mathcal {P}$ come from simpler spaces $S_{\underline {\mu }}(V)$ for finitely many tuples $\underline {\mu }\lessdot \underline {\lambda }$.

We define later what it means to “come from $S_{\underline {\mu }}(V)$”; for a more precise formulation of the theorem, see Theorem 2.5.2. When $\underline {\lambda }$ consists of one partition, the second case in the theorem says that elements satisfying $\mathcal {P}$ have bounded strength in the following sense.

### **Definition 1**

The strength of an element *f* ∈ *S*_*λ*_(*V* ) with *λ* ⊩ *d* is the minimal integer *k* ≥ 0 such that there exists an expression

$$ f= \alpha_{1}(g_{1}, h_{1})+\cdots+ \alpha_{k}(g_{k}, h_{k}), $$

where *μ*_*i*_ ⊩ *d*_*i*_, *ν*_*i*_ ⊩ *e*_*i*_ with *d*_*i*_,*e*_*i*_ < *d*, the $\alpha _{i} \colon S_{\mu _{i}}(V)\oplus S_{\nu _{i}}(V)\to S_{\lambda }(V)$ are GL(*V* )-equivariant bilinear maps and the $g_{i}\in S_{\mu _{i}}(V)$, $h_{i}\in S_{\nu _{i}}(V)$ are elements.

In Definition 2.2.6 we will give a broader definition that is equivalent to the one above for tuples consisting of a single partition. The definition above and Definition 2.2.6 extend the strength of polynomials and of tuples of polynomials, respectively. Strength of polynomials plays a key role in the resolution of Stillman’s conjecture by Ananyan–Hochster [1] and in recent work by Kazhdan–Ziegler [19, 20]. Main Theorem I is an extension (in characteristic zero) of [20, Theorem 1.9] for homogeneous polynomials, which is the case where $\underline {\lambda }$ is a single partition with a single row.

Next, denote the inverse limit of the spaces $S_{\underline {\lambda }}(K^{n})$ mapping to each other via $S_{\underline {\lambda }}$ applied to the projection maps *K*^*n*+ 1^ → *K*^*n*^ by $S_{\underline {\lambda },\infty }$. This space comes with the action of the direct limit $\text {GL}_{\infty }$ of the groups GL_*n*_ mapping into each other via the maps *g*↦diag(*g*,1). It also comes with a topology induced by the Zariski topologies on $S_{\underline {\lambda }}(K^{n})$, which we again call the Zariski topology.

### **Corollary 1** (Corollary 2.6.3)

Suppose that the orbit $\text {GL}_{\infty } \cdot p$ is Zariski dense in $S_{\underline {\lambda },\infty }$. Then for each integer *n* ≥ 1, the image of $\text {GL}_{\infty } \cdot p$ in $S_{\underline {\lambda }}(K^{n})$ is all of $S_{\underline {\lambda }}(K^{n})$.

The second goal of this paper is to bring some order in the (typically uncountable) set of elements with dense $\text {GL}_{\infty }$-orbits. For elements $p,q\in S_{\underline {\lambda },\infty }$, we write *p* ≼ *q* when *q* specialises to *p*; see Sections 2.7–2.8 for details.

### *Example 3*

When *λ* = (*d*) ⊩ *d*, the space $S_{\lambda ,\infty }$ consists of infinite degree-*d* forms in variables *x*_1_,*x*_2_,…. We have *p* ≼ *q* if and only if *p* = *q*(*ℓ*_1_,*ℓ*_2_,…) where *ℓ*_1_,*ℓ*_2_,… are infinite linear forms such that for all *i* ≥ 1, the variable *x*_*i*_ occurs in only finitely many forms *ℓ*_*j*_; this ensures that *q*(*ℓ*_1_,*ℓ*_2_,…) is a well-defined infinite form of degree *d*.

Our second main result is the following theorem.

### **Main Theorem II 1** (Theorem 2.9.1)

Let $\underline {\lambda }$ be a tuple of partitions, all of the same integer *d* ≥ 1. There exist elements $p,r\in S_{\underline {\lambda },\infty }$, each with a dense $\text {GL}_{\infty }$-orbit, such that *p* ≼ *q* ≼ *r* for all other $q\in S_{\underline {\lambda },\infty }$ with a dense $\text {GL}_{\infty }$-orbit.

### Structure of the paper

In Section 2, we introduce all relevant definitions and restate our main results in more precise terms. Also, while our main results require characteristic zero, some of our theory is developed in arbitrary characteristic. In Section 3, we prove Main Theorem I. In Section 4, we prove Main Theorem II by constructing minimal *p* and maximal *r*. Finally, we end with some examples in Section 5.

## Definitions and Main Results

Fix a field *K*. In our main results we will assume that *K* is algebraically closed and of characteristic zero, but for now we make no such assumption.

### Strength

#### **Definition 2.1.1**

Let *n* ≥ 1 be an integer and let *f* ∈ *K*[*x*_1_,…,*x*_*n*_]_*d*_ be a homogeneous polynomial of degree *d* ≥ 2. Then the *strength* of *f*, denoted str(*f*), is the minimal integer *k* ≥ 0 such that there exists an expression

$$ f= g_{1}\cdot h_{1}+\cdots+ g_{k}\cdot h_{k}, $$

where $g_{i} \in K[x_{1},\ldots ,x_{n}]_{d_{i}}$ and $h_{i} \in K[x_{1},\ldots ,x_{n}]_{d-d_{i}}$ for some integer 0 < *d*_*i*_ < *d* for each *i* ∈ [*k*].

The strength of polynomials plays a key role in the resolution of Stillman’s conjecture by Ananyan–Hochster [1, 2], the subsequent work by Erman–Sam–Snowden [12–14] and in Kazhdan–Ziegler’s work [19, 20]. Also see [3–5, 7, 9, 10] for other recent papers studying strength.

### Polynomial Functors and Their Maps

Assume that *K* is infinite. Let **Vec** be the category of finite-dimensional vector spaces over *K* with *K*-linear maps.

#### **Definition 2.2.1**

A *polynomial functor of degree* ≤ *d* over *K* is a functor *P* : **Vec** →**Vec** with the property that for all *U*, *V* ∈**Vec** the map *P* : Hom(*U*, *V* ) →Hom(*P*(*U*),*P*(*V* )) is a polynomial map of degree ≤ *d*. A *polynomial functor* is a polynomial functor of degree ≤ *d* for some integer $d<\infty $.

#### *Remark 2.2.2*

For finite fields *K*, the correct analogue is that of a *strict* polynomial functor [15].

Any polynomial functor *P* is a finite direct sum of its *homogeneous* parts *P*_*d*_, which are the polynomial subfunctors defined by *P*_*d*_(*V* ) := {*p* ∈ *P*(*V* )∣∀*t* ∈ *K* : *P*(*t* id_*V*_)*p* = *t*^*d*^*p*} for each integer *d* ≥ 0. A polynomial functor is called homogeneous of degree *d* when it equals its degree-*d* part.

#### *Example 2.2.3*

The functor *U*↦*S*^*d*^(*U*) is a homogeneous polynomial functor of degree *d*. If *U* has basis *x*_1_,…,*x*_*n*_, then *S*^*d*^(*U*) is canonically isomorphic to *K*[*x*_1_,…,*x*_*n*_]_*d*_. In this incarnation, linear maps *S*^*d*^(*φ*) for *φ*: *U* → *V* correspond to substitutions of the variables *x*_1_,…,*x*_*n*_ by linear forms in variables *y*_1_,…,*y*_*m*_ representing a basis of *V*.

Polynomial functors are the ambient spaces in current research on infinite-dimensional algebraic geometry [6–8, 11]. Polynomial functors form an Abelian category in which a morphism *α*: *P* → *Q* consists of a linear map *α*_*U*_: *P*(*U*) → *Q*(*U*) for each *U* ∈**Vec** such that for all *U*, *V* ∈**Vec** and all *φ* ∈Hom(*U*, *V* ) the following diagram commutes: 
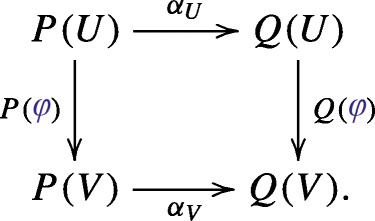
 In characteristic zero, each polynomial functor *P* is isomorphic, in this Abelian category, to a direct sum of Schur functors, which can be thought of as subobjects (or quotients) of the polynomial functors *V* ↦*V*^⊗*d*^. For that reason, we will informally refer to elements of*P*(*V* ) as*tensors*.

In addition to the linear morphisms between polynomial functors above, we may also allow each *α*_*U*_ to be a *polynomial* map *P*(*U*) → *Q*(*U*) such that the diagram commutes. Such an *α* will be called a *polynomial transformation* from *P* to *Q*. If *U* is irrelevant or clear from the context, we write *α* instead of *α*_*U*_.

#### *Example 2.2.4*

In the context of Definition 2.1.1, we set $P:=\bigoplus _{i=1}^{k} (S^{d_{i}} \oplus S^{d-d_{i}})$ and *Q* := *S*^*d*^ and define *α* by

$$ \alpha(g_{1},h_{1},\ldots,g_{k},h_{k}):=g_{1}\cdot h_{1} + {\cdots} + g_{k}\cdot h_{k}. $$

This is a polynomial transformation *P* → *Q*.

#### *Example 2.2.5*

Let *Q*, *R* be polynomial functors and *α*: *Q* ⊗ *R* → *P* a linear morphism. Then (*q*, *r*)↦*α*(*q* ⊗ *r*) defines a *bilinear* polynomial transformation *Q* ⊕ *R* → *P*.

Inspired by these examples, we propose the following definition of strength for elements of homogeneous polynomial functors. We are not sure that this is the best definition in arbitrary characteristic, so we restrict ourselves to characteristic zero.

#### **Definition 2.2.6**

Assume that char*K* = 0. Let *P* be a homogeneous polynomial functor of degree *d* ≥ 2 and let *V* ∈**Vec**. The *strength* of *p* ∈ *P*(*V* ) is the minimal integer *k* ≥ 0 such that

$$ p=\alpha_{1}(q_{1},r_{1})+\cdots+\alpha_{k}(q_{k},r_{k}) $$

where, for each *i* ∈ [*k*], *Q*_*i*_, *R*_*i*_ are irreducible polynomial functors with positive degrees adding up to *d*, *α*_*i*_: *Q*_*i*_ ⊕ *R*_*i*_ → *P* is a bilinear polynomial transformation and *q*_*i*_ ∈ *Q*_*i*_(*V* ) and *r*_*i*_ ∈ *R*_*i*_(*V* ) are tensors.

#### *Remark 2.2.7*

Positive degrees of two polynomial functors cannot add up to 1. So nonzero tensors *p* ∈ *P*(*V* ) of homogeneous polynomial functors *P* of degree 1 cannot have finite strength. We say that such tensors *p* have infinite strength. Note that the strength of 0 ∈ *P*(*V* ) always equals 0.

#### **Proposition 2.2.8**

Assume that char*K* = 0. For each integer *d* ≥ 2, the strength of a polynomial *f* ∈ *S*^*d*^(*V* ) according to Definition 2.1.1 equals that according to Definition 2.2.6.

#### *Proof*

The inequality ≥ follows from the fact that $\alpha _{i}\colon S^{d_{i}} \oplus S^{d-d_{i}} \to S^{d},(g,h)\mapsto g\cdot h$ is a bilinear polynomial transformation. For the inequality ≤, suppose that *α*: *Q* ⊕ *R* → *S*^*d*^ is a nonzero bilinear polynomial transformation, where *Q* and *R* are irreducible of degrees *e* < *d* and *d* − *e* < *d*. So *Q* and *R* are Schur functors corresponding to Young diagrams with *e* and *d* − *e* boxes, respectively, and *Q* ⊗ *R* admits a nonzero linear morphism to *S*^*d*^, whose Young diagram is a row of *d* boxes. The Littlewood–Richardson rule then implies that the Young diagrams of *Q* and *R* must be a single row as well, so that *Q* = *S*^*e*^ and *R* = *S*^*d*−*e*^, and also that there is (up to scaling) a unique morphism *Q* ⊗ *R* = *S*^*e*^ ⊗ *S*^*d*−*e*^ → *S*^*d*^, namely, the one corresponding to the polynomial transformation (*g*, *h*)↦*g* ⋅ *h*. □

The strength of a tensor in *P* quickly becomes very difficult when *P* is not irreducible.

#### *Example 2.2.9*

Take *P* = (*S*^*d*^)^⊕*e*^ for some integer *e* ≥ 1. The strength of a tuple (*f*_1_,…,*f*_*e*_) ∈ *P*(*V* ) is the minimum number *k* ≥ 0 such that

$$ f_{1},\ldots,f_{e}\in\text{span}\{g_{1},\ldots,g_{k}\}, $$

where *g*_1_,…,*g*_*k*_ ∈ *S*^*d*^(*V* ) are reducible polynomials.

#### *Example 2.2.10*

Consider $P=S^{2}\oplus \bigwedge ^{2}$, so that *P*(*V* ) = *V* ⊗ *V*, and assume that *K* is algebraically closed. The only possibilities for *Q* and *R* are *Q*(*V* ) = *R*(*V* ) = *V*. The bilinear polynomial transformations *α* : *Q* ⊕ *R* → *P* are of the form

$$ \alpha(u,v)=a u \otimes v + b v \otimes u=c(u\otimes v+v\otimes u)+d(u\otimes v-v\otimes u) $$

for certain *a*, *b*, *c*, *d* ∈ *K*. We note that str(*A*) = ⌈rk(*A*)/2⌉ when *A* ∈ *S*^2^(*V* ) and str(*A*) = rk(*A*)/2 when $A\in \bigwedge ^{2}(V)$. In general, we have

$$ \text{rk}(A)/2,\text{rk}(A+A^{\top})/2,\text{rk}(A-A^{\top})/2\leq \text{str}(A)\leq \text{rk}(A),\text{rk}(A+A^{\top})/2+\text{rk}(A-A^{\top})/2 $$

for all *A* ∈ *V* ⊗ *V*, where each bound can hold with equality. For example, for the matrix

$$ A=\begin{pmatrix} 0&1\\0&0\\&&\ddots\\&&&0&1\\&&&0&0 \end{pmatrix} $$

we have rk(*A* + *A*^⊤^)/2 = rk(*A* − *A*^⊤^)/2 = str(*A*) = rk(*A*).

#### *Example 2.2.11*

Again take $P=S^{2}\oplus \bigwedge ^{2}$ and consider *P*(*K*^2^) = *K*^2×2^. Assume *K* is algebraically closed. The matrix

$$ A =\begin{pmatrix}1&x\\0&1\end{pmatrix} $$

clearly has strength ≤ 2. We will show that *A* has strength 2 whenever *x* = ± 2 and strength 1 otherwise. In particular, this shows that the subset of *P*(*K*^2^) of matrices of strength ≤ 1 is not closed.

Suppose *A* has strength 1. Then we can write *A* as *au* ⊗ *v* + *bv* ⊗ *u* with *a*, *b* ∈ *K* and *v*, *u* ∈ *K*^2^. Let *e*_1_, *e*_2_ be the standard basis of *K*^2^. Without loss of generality, we may assume that *u* = *e*_1_ + *λe*_2_ and *v* = *e*_1_ + *μe*_2_ for some *λ*, *μ* ∈ *K*. We get

$$ \begin{array}{rlrl} a+b&=1,\qquad&a\mu+b\lambda&=x,\\ a\lambda+b\mu&=0,\qquad&\lambda\mu&=1. \end{array} $$

Using *λ* = *μ*^− 1^ and *b* = 1 − *a*, we are left with *aμ*^2^ + (1 − *a*) = *xμ* and *a* + (1 − *a*)*μ*^2^ = 0. The latter gives us *μ*≠ ± 1 and *a* = *μ*^2^/(*μ*^2^ − 1). We get *μ*^2^ + 1 = *xμ*. Now, if *x*≠ ± 2, then such a *μ*≠ ± 1 exists. So in this case *A* indeed has strength 1. If *x* = ± 2, the only solution is *μ* = ± 1. Hence *A* has strength 2 in this case.

### Subsets of Polynomial Functors

#### **Definition 2.3.1**

Let *P* be a polynomial functor. A *subset* of *P* consists of a subset $X(U) \subseteq P(U)$ for each *U* ∈**Vec** such that for all *ϕ* ∈Hom(*U*, *V* ) we have $P(\varphi )(X(U)) \subseteq X(V)$. It is *closed* if each *X*(*U*) is Zariski-closed in *P*(*U*).

#### *Example 2.3.2*

Fix integers *d* ≥ 2 and *k* ≥ 0. The elements in *S*^*d*^(*V* ) of strength ≤ *k* form a subset of *S*^*d*^. This set is closed for *d* = 2,3 but not for *d* = 4; see [3].

#### *Example 2.3.3*

Take $K=\mathbb {R}$ and let *X*(*V* ) be the set of positive semidefinite elements in *S*^2^(*V* ), i.e., those that are sums of squares of elements of *V*. Then *X* is a subset of *S*^2^.

### Kazhdan–Ziegler’s Theorem: Universality of Strength

#### **Theorem 2.4.1** (Kazhdan–Ziegler (20, Theorem 1.9))

Let *d* ≥ 2 be an integer. Assume that *K* is algebraically closed and of characteristic 0 or > *d*. Let *X* be a subset of *S*^*d*^. Then either *X* = *S*^*d*^ or else there exists an integer *k* ≥ 0 such that each polynomial in each *X*(*U*) has strength ≤ *k*.

This theorem is a strengthening of [7, Theorem 4], where the additional assumption is that *X* is closed. The condition that *K* be algebraically closed cannot be dropped, e.g. by Example 2.3.3: there is no uniform upper bound on the strength of positive definite quadratic forms. The condition on the characteristic can also not be dropped, but see Remark 2.9.2.

#### **Corollary 2.4.2** (Kazhdan–Ziegler, universality of strength)

With the same assumptions on *K*, for every fixed number of variables *m* ≥ 1 and degree *d* ≥ 2 there exists an *r* ≥ 0 such that for any number of variables *n* ≥ 1, any polynomial *f* ∈ *K*[*x*_1_,…,*x*_*n*_]_*d*_ of strength ≥ *r* and any polynomial *g* ∈ *K*[*y*_1_,…,*y*_*m*_]_*d*_ there exists a linear variable substitution $x_{j} \mapsto {\sum }_{i} c_{ij} y_{i}$ under which *f* specialises to *g*.

#### *Proof*

For each *U* ∈**Vec**, define $X(U) \subseteq S^{d}(U)$ as the set of all *f* such that the map

$$ \begin{array}{@{}rcl@{}} \text{Hom}(U,K^{m}) &\to& S^{d}(K^{m})\\ \phi &\mapsto& S^{d}(\phi)f \end{array} $$

is *not* surjective. A straightforward computation shows that this is a subset of *S*^*d*^. It is not all of *S*^*d*^, because if we take *U* to be of dimension $d \cdot \dim S^{d}(K^{m})$, then in *S*^*d*^(*U*) we can construct a sum *f* of $\dim S^{d}(K^{m})$ squarefree monomials in distinct variables and specialise each of these monomials to a prescribed multiple of a basis monomial in *S*^*d*^(*K*^*m*^). Hence *f*∉*X*(*U*). By Theorem 2.4.1, it follows that the strength of elements of *X*(*U*) is uniformly bounded. □

### Our Generalisation: Universality for Polynomial Functors

Let *P*, *Q* be polynomial functors. We say that *Q* is smaller than *P*, denoted $Q\lessdot P$, when *P* and *Q* are not (linearly) isomorphic and *Q*_*d*_ is a quotient of *P*_*d*_ for the highest degree *d* where *P*_*d*_ and *Q*_*d*_ are not isomorphic. We say that a polynomial functor *P* is pure when *P*({0}) = {0}.

#### *Remark 2.5.1*

Let $Q\lessdot P$ be polynomial functors and suppose that *P* is homogeneous of degree *d* > 0. Then *Q*_*d*_ must be a quotient of *P*_*d*_. So we see that $Q\oplus R\lessdot P$ for any polynomial functor *R* of degree < *d*.

The following is our first main result.

#### **Theorem 2.5.2** (Main Theorem I)

Assume that *K* is algebraically closed of characteristic zero. Let *X* be a subset of a pure polynomial functor *P* over *K*. Then either *X*(*U*) = *P*(*U*) for all *U* ∈**Vec** or else there exist finitely many polynomial functors $Q_{1},\ldots ,Q_{k}\lessdot P$ and polynomial transformations *α*_*i*_: *Q*_*i*_ → *P* with $X(U) \subseteq \bigcup _{i=1}^{k} \text {im}(\alpha _{i,U})$ for all *U* ∈**Vec**. In the latter case, *X* is contained in a proper closed subset of *P*.

If we assume furthermore that *P* is irreducible, then in the second case there exists a integer *k* ≥ 0 such that for all *U* ∈**Vec** and all *p* ∈ *X*(*U*) the strength of *p* is at most *k*.

This is a strengthening of a theorem from the upcoming paper [8] (also appearing in the first author’s thesis [6, Theorem 4.2.5]), where the additional assumption is that *X* be closed.

#### *Remark 2.5.3*

When *P* is irreducible of degree 1, then *P*(*U*) = *U*. In this case, the subsets of *P* are *P* and {0}. So indeed, the elements of a proper subset of *P* have bounded strength, namely 0.

Again, the condition that *K* be algebraically closed cannot be dropped, and neither can the condition on the characteristic; however, see Remark 2.9.2. Main Theorem I has the same corollary as Theorem 2.4.1.

#### **Corollary 2.5.4**

With the same assumptions as in Main Theorem I, let *U* ∈**Vec** be a fixed vector space. Then there exist finitely many polynomial functors $Q_{1},\ldots ,Q_{k}\lessdot P$ and polynomial transformations *α*_*i*_: *Q*_*i*_ → *P* such that for every *V* ∈**Vec** and every *f* ∈ *P*(*V* ) that is *not* in $\bigcup _{i=1}^{k} \text {im}(\alpha _{i,V})$ the map Hom(*V*, *U*) → *P*(*U*),*φ*↦*P*(*φ*)*f* is surjective.

If *P* is irreducible, then the condition that $f \not \in \bigcup _{i=1}^{k} \text {im}(\alpha _{i,V})$ can be replaced by the condition that *f* has strength greater than some function of $\dim U$ only.

### Limits and Dense Orbits

Let *P* be a pure polynomial functor over *K*. There is another point of view on closed subsets of *P*, which involves limits that we define now.

#### **Definition 2.6.1**

We define $P_{\infty }:=\varprojlim _{n} P(K^{n})$, where the map *P*(*K*^*n*+ 1^) → *P*(*K*^*n*^) is *P*(*π*_*n*_) with *π*_*n*_: *K*^*n*+ 1^ → *K*^*n*^ the projection map forgetting the last coordinate. We equip $P_{\infty }$ with the inverse limit of the Zariski topologies on the *P*(*K*^*n*^), which is itself a Zariski topology coming from the fact that $P_{\infty }=(\bigcup _{n} P(K^{n})^{\ast })^{\ast }$. We also write *P*(*π*_*n*_) for the projection map $P_{\infty } \to P(K^{n})$; this will not lead to confusion. A polynomial transformation *α*: *P* → *Q* naturally yields a continuous map $P_{\infty } \to Q_{\infty }$ also denoted by *α*.

If *P* = *S*^*d*^, then the elements of $P_{\infty }$ can be thought of as homogeneous series of degree *d* in infinitely many variables *x*_1_,*x*_2_,…. Here, closed subsets of $P_{\infty }$ are defined by polynomial equations in the coefficients of these series.

On $P_{\infty }$ acts the group $\text {GL}_{\infty }=\bigcup _{n} \text {GL}_{n}$, where GL_*n*_ is embedded into GL_*n*+ 1_ via the map

$$ g \mapsto \begin{pmatrix} g & 0 \\ 0 & 1 \end{pmatrix}. $$

Indeed, with this embedding the map *P*(*K*^*n*+ 1^) → *P*(*K*^*n*^) in the definition of $P_{\infty }$ is GL_*n*_-equivariant, and this yields the action of $\text {GL}_{\infty }$ on the projective limit. In the case of degree-*d* series, an element $g\in \text {GL}_{n}\subset \text {GL}_{\infty }$ maps each of the first *n* variables *x*_*i*_ to a linear combination of *x*_1_,…,*x*_*n*_ and the remaining variables to themselves.

The map that sends a closed subset *X* of *P* to the closed subset $X_{\infty }:= \varprojlim _{n} X(K^{n})$ of $P_{\infty }$ is a bijection with the collection of closed $\text {GL}_{\infty }$-stable subsets of $P_{\infty }$ [6, Proposition 1.3.28]. Hence closed subsets of polynomial functors can also be studied in this infinite-dimensional setting.

#### *Example 2.6.2*

On degree-*d* forms, $\text {GL}_{\infty }$ clearly has dense orbits, such as that of

$$ f=x_{1} x_{2} {\cdots} x_{d} + x_{d+1} x_{d+2} {\cdots} x_{2d} + \cdots $$

The reason is that this series can be specialised to any degree-*d* form *in finitely many variables* by linear variable substitutions. This implies that the image of $\text {GL}_{\infty } \cdot f$ in each *S*^*d*^(*K*^*n*^) is dense. Hence $\text {GL}_{\infty }\!\cdot f$ is dense in $S^{d}_{\infty }$.

For every pure polynomial functor *P*, the group $\text {GL}_{\infty }$ has dense orbits on $P_{\infty }$—in fact, uncountably many of them! See [6, §4.5.1]. They have the following interesting property.

#### **Corollary 2.6.3**

Suppose that $\text {GL}_{\infty } \cdot p$ is dense in $P_{\infty }$. Then for each integer *n* ≥ 1, the image of $\text {GL}_{\infty }\!\cdot p$ in *P*(*K*^*n*^) is all of *P*(*K*^*n*^).

#### *Proof*

For *V* ∈**Vec**, define

$$ X(V):= \left\{P(\varphi)P(\pi_{n})p ~|~ n\geq 1, \varphi\in\text{Hom}(K^{n},V)\right\}\subseteq P(V), $$

which is exactly the image of $\text {GL}_{\infty }\!\cdot p$ under the projection $P_{\infty } \to P(K^{m})$ followed by an isomorphism *P*(*φ*), where *φ*: *K*^*m*^ → *V* is a linear isomorphism. We see that *X* is a subset of *P*. For each *V* ∈**Vec**, the subset *X*(*V* ) is dense in *P*(*V* ) since $\text {GL}_{\infty }\!\cdot p$ is dense in $P_{\infty }$. So *X* = *P* by Main Theorem I. □

The notion of strength has an obvious generalisation.

#### **Definition 2.6.4**

Assume that char*K* = 0. Let *P* be a homogeneous polynomial functor. The strength of a tensor $p \in P_{\infty }$ is the minimal integer *k* ≥ 0 such that

$$ p=\alpha_{1}(q_{1},r_{1})+\cdots+\alpha_{k}(q_{k},r_{k}) $$

for some irreducible polynomial functors *Q*_*i*_, *R*_*i*_ whose positive degrees sum up to *d*, bilinear polynomial transformations *α*_*i*_: *Q*_*i*_ ⊕ *R*_*i*_ → *P* and elements $q_{i} \in Q_{i,\infty }$ and $r_{i} \in R_{i,\infty }$. If no such *k* exists, we say that *p* has infinite strength.

#### **Corollary 2.6.5**

Assume that char*K* = 0 and that *P* is irreducible of degree ≥ 2. Then an element of $P_{\infty }$ has infinite strength if and only if its $\text {GL}_{\infty }$-orbit is dense.

#### *Proof*

If $p \in P_{\infty }$ has finite strength, then let *α*_*i*_: *Q*_*i*_ × *R*_*i*_ → *P* be as in the definition above and let

$$ \alpha:=\alpha_{1} +{\cdots} + \alpha_{k}\colon Q:=\bigoplus\limits_{i=1}^{k} (Q_{i} \otimes R_{i}) \to P $$

be their sum, so that *p* ∈im(*α*). Consider the closed subset $X=\overline {\text {im}(\alpha )}$, i.e., the closed subset defined by $X(V)=\overline {\text {im}(\alpha _{V})}$ for all *V* ∈**Vec**. As $\dim Q(K^{n})$ is a polynomial in *n* of degree < *d*, while $\dim P(K^{n})$ is a polynomial in *n* of degree *d*, we see that *X*(*K*^*n*^) is a proper subset of *P*(*K*^*n*^) for all *n* ≫ 0. Since $p\in X_{\infty }$, it follows that $\text {GL}_{\infty }\!\cdot p$ is not dense.

Suppose, conversely, that $\text {GL}_{\infty }\!\cdot p$ is not dense. Then it is contained in $X_{\infty }$ for some proper closed subset *X* of *P*. Hence *p* has finite strength by Main Theorem I. □

#### *Example 2.6.6*

Let *P*, *Q* be homogeneous functors of the same degree *d* ≥ 2 and let $p\in P_{\infty }$ be an element of infinite strength. Then $(p,0)\in P_{\infty }\oplus Q_{\infty }$ also has infinite strength, but the orbit $\text {GL}_{\infty }\!\cdot (p,0)$ is not dense.

#### *Remark 2.6.7*

In Section 4 we will use a generalisation of notation introduced here: for an integer *m* ≥ 0 we will write $P_{\infty -m}$ for the limit $\varprojlim _{n} P(K^{[n]-[m]})$ over all integers *n* ≥ *m*. This space is isomorphic to $P_{\infty }$, but the indices have been shifted by *m*. On $P_{\infty - m}$ acts the group $\text {GL}_{\infty -m} \cong \text {GL}_{\infty }$, which is the union of GL(*K*^[*n*]−[*m*]^) over all *n* ≥ *m*. We denote the image of an element $p\in P_{\infty -m}$ in *P*(*K*^[*n*]−[*m*]^) by *p*_[*n*]−[*m*]_. The inclusions *ι*_*n*_: *K*^[*n*]−[*m*]^ → *K*^*n*^ sending *v*↦(0,*v*) allow us to view $P_{\infty -m}$ as a subset of $P_{\infty }$.

#### **Corollary 2.6.8**

Let *P* be a homogeneous polynomial functor of degree *d* ≥ 2 and *m* ≥ 0 an integer. Let $p\in P_{\infty -m}$ be a tensor whose $\text {GL}_{\infty -m}$-orbit is not dense and let $q\in P_{\infty }$ be an element with finite strength. Then the $\text {GL}_{\infty }$-orbit of $p+q\in P_{\infty }$ is also not dense.

#### *Proof*

Note that *p* is contained in the image of $\alpha \colon Q_{\infty -m} \to P_{\infty -m}$ for some polynomial transformation *α*: *Q* → *P* with $Q\lessdot P$ [6, Theorem 4.2.5] and *q* is contained in the image of $\beta \colon R_{\infty } \to P_{\infty }$ for some polynomial transformation *β*: *R* → *P* with deg(*R*) < *d*. So since $Q\oplus R\lessdot P$ by Remark 2.5.1, we see that *p* + *q* is contained in a proper closed subset of *P*. Hence its $\text {GL}_{\infty }$-orbit is not dense. □

### Linear Endomorphisms

Our second goal in this paper is to show that there always exist *minimal* *f* with dense orbits. This minimality relates to a monoid of linear endomorphisms extending $\text {GL}_{\infty }$, as follows. Elements of $\text {GL}_{\infty }$ are $\mathbb {N}\times \mathbb {N}$ matrices of the block form

$$ \begin{pmatrix} g & 0 \\ 0 & I_{\infty} \end{pmatrix}, $$

where *g* ∈GL_*n*_ for some *n* and $I_{\infty }$ is the infinite identity matrix.

#### **Definition 2.7.1**

Let $E \supset \text {GL}_{\infty }$ be the monoid of $\mathbb {N} \times \mathbb {N}$ matrices with the property that each *row* contains only finitely many nonzero entries.

#### *Example 2.7.2*

For every integer *i* ≥ 1, let $\varphi _{i}\in K^{n_{i}\times m_{i}}$ be a matrix. Then the block matrix

$$ \begin{pmatrix} \varphi_{1}\\&\varphi_{2}\\&&\ddots \end{pmatrix} $$

is an element of *E*.

We define an action of *E* on $P_{\infty }$ as follows. Let $p=(p_{0},p_{1},\ldots ) \in P_{\infty }$ and *φ* ∈ *E*. For each integer *i* ≥ 0, to compute *q*_*i*_ in

$$ q=(q_{0},q_{1},\ldots) =P(\varphi) p $$

we choose *n*_*i*_ ≥ 0 such that all the nonzero entries of the first *i* rows of *φ* are in the first *n*_*i*_ columns. Now, we let $\psi _{i} \in K^{i\times n_{i}}$ be the *i* × *n*_*i*_ block in the upper-left corner of *φ*, so that

$$ \varphi=\begin{pmatrix} \psi_{i} & 0 \\ \ast & \ast \end{pmatrix}, $$

and we set $q_{i}:=P(\psi _{i})p_{n_{i}}$. Note that if we replace *n*_*i*_ by a larger number $\tilde {n}_{i}$, then the resulting matrix $\tilde {\psi }_{i}$ satisfies $\tilde {\psi }_{i} = \psi _{i} \circ \pi $, where $\pi :K^{\tilde {n}_{i}} \to K^{n_{i}}$ is the projection. Consequently, we then have

$$ P(\tilde{\psi}_{i}) p_{\tilde{n}_{i}}=P(\psi_{i})P(\pi)p_{\tilde{n}_{i}} = P(\psi_{i}) p_{n_{i}}, $$

so that *q*_*i*_ is, indeed, well-defined. A straightforward computation shows that, for *φ*, *ψ* ∈ *E*, we have *P*(*ψ*) ∘ *P*(*φ*) = *P*(*ψ* ∘ *φ*), so that *E* does indeed act on $P_{\infty }$.

For infinite degree-*d* forms, the action of *φ* ∈ *E* is by linear variable substitutions $x_{j} \mapsto {\sum }_{i=1}^{\infty } \varphi _{ij} x_{i}$. Note that, since each *x*_*i*_ appears in the image of only finitely many *x*_*j*_, this substitution does indeed make sense on infinite degree-*d* series.

Since $\text {GL}_{\infty } \subseteq E$, an *E*-stable subset of $P_{\infty }$ is also $\text {GL}_{\infty }$-stable. The converse does not hold, since for instance *E* also contains the zero matrix, and *P*(0)*f* = 0≠*P*(*g*)*f* for all nonzero $f\in P_{\infty }$ and $g \in \text {GL}_{\infty }$ when the polynomial functor *P* is pure. However, it is easy to see that $\text {GL}_{\infty }$-stable *closed* subsets of $P_{\infty }$ are also *E*-stable. In particular, we have $\overline {\text {GL}_{\infty }\!\cdot f}=\overline {P(E)f}$.

### A Quasi-order on Infinite Tensors

#### **Definition 2.8.1**

For infinite tensors $p,q \in P_{\infty }$ we write *p* ≼ *q* if *p* ∈ *P*(*E*)*q*. In this case, we say that *q* *specialises* to *p*.

From the fact that *E* is a unital monoid that acts on $P_{\infty }$, we find that ≼ is transitive and reflexive. Hence, it induces an equivalence relation $\simeq $ on $P_{\infty }$ by

$$ p \simeq q :~\Leftrightarrow~p \preceq q \text{ and } q \preceq p, $$

as well as a partial order on the equivalence classes of $\simeq $.

#### *Example 2.8.2*

Fix an integer *k* ≥ 1 and consider the polynomial functor *P* = (*S*^1^)^⊕*k*^. A tuple $q=(q_{1},\ldots ,q_{k})\in P_{\infty }$ has a dense $\text {GL}_{\infty }$-orbit if and only if $q_{1},\ldots ,q_{k}\in S^{1}_{\infty }$ are linearly independent. Suppose that *q* has a dense $\text {GL}_{\infty }$-orbit and let *A* be the $\mathbb {N} \times k$ matrix corresponding to *q*. Then *A* has full rank. By acting with an element of $\text {GL}_{\infty }\subseteq E$, we may assume that

$$ A=\binom{I_{k}}{B}, $$

where *B* is again an $\mathbb {N}\times k$ matrix. Now, take

$$ \varphi_{C}:=\begin{pmatrix} I_{k}\\C&I_{\infty} \end{pmatrix}\in E $$

and note that *ϕ*_−*B*_*A* = (*I*_*k*_ 0)^⊤^, so that *P*(*ϕ*_−*B*_)*q* = (*x*_1_,…,*x*_*k*_). So any two tuples in $P_{\infty }$ with a dense $\text {GL}_{\infty }$-orbit are in the same equivalence class. Moreover, the element of *E* specialising one tuple to the other can be chosen to be invertible in *E* as $\phi _{C}\varphi _{-C}=I_{\infty }$.

There is an obvious relation between ≼ and orbit closures, namely: if *p* ≼ *q*, then $p \in \overline {\text {GL}_{\infty }\!\cdot q}$. The converse, however, is not true.

#### *Example 2.8.3*

Let $p=x_{1}({x_{1}^{2}}+{x_{2}^{2}}+\cdots ),q={x_{1}^{3}}+{x_{2}^{3}}+\cdots \in S^{3}_{\infty }$. Then *q* has infinite strength and so $p\in S^{3}_{\infty }=\overline {\text {GL}_{\infty }\!\cdot q}$. However, we have *p*⋠*q*: suppose that

$$ f := x_{1}g(x_{1},x_{2},\ldots)+h(x_{2},x_{3},\ldots)\in S^{3}(E)q $$

for some $g\in S^{2}_{\infty }$ and $h\in S^{3}_{\infty }$. As only finitely many variables *x*_*i*_ are substituted by linear forms containing *x*_1_ when specialising *q* to *f*, we see that

$$ x_{1}g(x_{1},x_{2},\ldots)+\tilde{h}(x_{2},x_{3},\ldots)\in S^{3}(E)({x_{1}^{3}}+{x_{2}^{3}}+\cdots+{x_{n}^{3}}) $$

for some integer *n* ≥ 1 and $\tilde {h}\in S^{3}_{\infty }$. From this, it is easy to see that *g* has finite strength. Hence *f*≠*p* as ${x_{1}^{2}}+{x_{2}^{2}}+\cdots $ has infinite strength. So indeed *p*⋠*q*.

In order to have a tensor $p\in P_{\infty }$ with a dense $\text {GL}_{\infty }$-orbit, the polynomial functor *P* must be pure. For some time, we believed that when this is the case all elements $p \in P_{\infty }$ with a dense $\text {GL}_{\infty }$-orbit might form a single $\simeq $-equivalence class. When *P* has degree ≤ 2, this is in fact true; see Example 5.1.4. However, it does not hold for cubics.

#### *Example 2.8.4*

Let $p,q\in S^{3}_{\infty }$ be as before. Now also consider *r* = *p*(*x*_1_,*x*_3_,…) + *q*(*x*_2_,*x*_4_,…). We have *q* = *r*(0,*x*_1_,0,*x*_2_,…) ≼ *r* and so $S^{3}_{\infty }=\overline {\text {GL}_{\infty }\!\cdot q}\subseteq \overline {\text {GL}_{\infty }\!\cdot r}$. Hence, both *q* and *r* have dense $\text {GL}_{\infty }$-orbits. And, we have *r*⋠*q*: indeed, otherwise *p* = *r*(*x*_1_,0,*x*_2_,0,…) ≼ *r* ≼ *q*, but *p*⋠*q*.

### Minimal Classes of Elements with Dense Orbits

Our second main result is the following.

#### **Theorem 2.9.1** (Main Theorem II)

Suppose that *K* is algebraically closed of characteristic zero. Let *P* be a pure homogeneous polynomial functor over *K*. Then there exist tensors $p,r \in P_{\infty }$ whose $\text {GL}_{\infty }$-orbits are dense such that *p* ≼ *q* ≼ *r* for all $q\in P_{\infty }$ whose $\text {GL}_{\infty }$-orbit is dense.

The elements *p* that have this property form a single $\simeq $-class which lies below the $\simeq $-classes of all other $q\in P_{\infty }$ whose $\text {GL}_{\infty }$-orbit is dense. For the construction of such a tensor $p\in P_{\infty }$, see Section 4.1. For the construction of the tensor $r \in P_{\infty }$, see Section 4.4.

#### *Remark 2.9.2*

In both our Main Theorems, we require that the characteristic be zero. This is because the results in [6] and [8] require this. However, the proof of topological Noetherianity for polynomial functors in [11] does not require characteristic zero, and shows that after a shift and a localisation, a closed subset of a polynomial functor admits a homeomorphism into an open subset of a smaller polynomial functor. In characteristic zero, this is in fact a closed embedding, so that it can be inverted and yields a parameterisation of (part of) the closed subset. In positive characteristic, it is not a closed embedding, but the map still becomes invertible if one formally inverts the Frobenius morphism; this is touched upon in [8]. This might imply variants of our Main Theorems in arbitrary characteristic, but we have not yet pursued this direction in detail.

## Proof of Main Theorem I

### The Linear Approximation of a Polynomial Functor

Let *P* be a polynomial functor over an infinite field and let *U*, *V* ∈**Vec**. Then $P(U \oplus V) = \bigoplus _{d,e=0}^{\infty } Q_{d,e}(U,V)$ where

$$ Q_{d,e}(U,V):=\{v \in P(U \oplus V) ~|~ \forall s,t \in K:~ P(s\text{id}_{U} \oplus t\text{id}_{V})v = s^{d} t^{e} v \}\subseteq P_{d+e}(U\oplus V). $$

The terms with *e* = 0 add up to *P*(*U*), and the terms with *e* = 1 add up to a polynomial bifunctor evaluated at (*U*, *V* ) that is linear in *V*. This is necessarily of the form $P^{\prime }(U) \otimes V$, where $P^{\prime }$ is a polynomial functor. In other words, we have

$$ P(U \oplus V)=P(U) \oplus (P^{\prime}(U) \otimes V) \oplus \text{higher-degree terms in }V. $$

We informally think of the first two terms as the linear approximation of *P* around *U*. Now suppose that we have a short exact sequence

$$ 0 \to P \to Q \to R \to 0 $$

of polynomial functors. This implies that for all *U*, *V* we have a short exact sequence

$$ \{0\} \to P(U \oplus V) \to Q(U \oplus V) \to R(U \oplus V) \to \{0\} $$

and inspecting the degree-1 parts in *V* we find a short exact sequence

$$ 0 \to P^{\prime} \to Q^{\prime} \to R^{\prime} \to 0. $$

This, and further straightforward computations, shows that $P \mapsto P^{\prime }$ is an exact functor from the category of polynomial functors to itself.

#### *Remark 3.1.1*

For *U* ∈**Vec** fixed, denote the polynomial functor sending *V* ↦*P*(*U* ⊕ *V* ) and *φ*↦*P*(id_*U*_ ⊕ *ϕ*) by Sh_*U*_(*P*). Then we have

$$ \text{Sh}_{U}(P)_{e}(V)=\{v \in P(U \oplus V) ~|~ \forall t \in K:~P(\text{id}_{U} \oplus t\text{id}_{V})v = t^{e} v \} $$

and from this we see that *Q*_*d*, *e*_(*U*, *V* ) = Sh_*U*_(*P*)_*e*_(*V* ) ∩ *P*_*d*+*e*_(*U* ⊕ *V* ). In particular, when *P* is homogeneous of degree *d*, we see that $P(U\oplus V)=\bigoplus _{e=0}^{d} Q_{d-e,e}(U,V)$ where *Q*_*d*−*e*, *e*_(*U*, *V* ) = Sh_*U*_(*P*)_*e*_(*V* ). Also note that, in this case, Sh_*U*_(*P*)_0_(*V* ) = *P*(*U*) and Sh_*U*_(*P*)_*d*_(*V* ) = *P*(*V* ) via the inclusions of *U*, *V* into *U* ⊕ *V*.

#### *Example 3.1.2*

If *P* = *S*^*d*^, then the formula

$$ S^{d}(U \oplus V) \cong \bigoplus_{e=0}^{d} S^{d-e}(U) \otimes S^{e}(V) = S^{d} (U) \oplus (S^{d-1} (U) \otimes V) \oplus\cdots $$

identifies $P^{\prime }$ with *S*^*d*− 1^.

#### *Example 3.1.3*

Let *K* be an algebraically closed field of characteristic *p*. Then *S*^*p*^ contains the subfunctor *P*(*V* ) := {*v*^*p*^∣*v* ∈ *V* }. We have *P*(*U* ⊕ *V* ) = *P*(*U*) ⊕ *P*(*V* ), and hence $P^{\prime }=0$.

### Proof of Main Theorem I

In this subsection we prove Theorem 2.5.2. We start with a result of independent interest.

#### **Theorem 3.2.1**

Let *P* be a pure polynomial functor over an algebraically closed field *K* of characteristic 0 or > deg(*P*) and let *X* be a subset of *P* such that *X*(*V* ) is dense in *P*(*V* ) for all *V* ∈**Vec**. Then, in fact, *X*(*V* ) is *equal* to *P*(*V* ) for all *V* ∈**Vec**.

Example 2.3.3 shows that the condition that *K* be algebraically closed cannot be dropped. We do not know if the condition on the characteristic of *K* can be dropped, but the proof will use thathe polynomial functor $P^{\prime }$ introduced in Section 3.1 is sufficiently large, which, by Example 3.1.3, need not be the case when char*K* is too small.

#### *Proof*

Let *q* ∈ *P*(*K*^*n*^). For each *k* ≥ *n*, we consider the incidence variety

$$ Z_{k}:=\{(\varphi,r) \in \text{Hom}(K^{k},K^{n}) \times P(K^{k}) ~|~ \text{rk}(\varphi)=n \text{ and } P(\varphi)r=q\}. $$

We write $e_{k}:=\dim _{K} P(K^{k})$. Since for every *φ* ∈Hom(*K*^*k*^,*K*^*n*^) of rank *n* the linear map *P*(*φ*) is surjective, *Z*_*k*_ is a vector bundle of rank *e*_*k*_ − *e*_*n*_ over the rank-*n* locus in Hom(*K*^*k*^,*K*^*n*^). Hence *Z*_*k*_ is an irreducible variety with $\dim Z_{k}=kn + e_{k}-e_{n}$. We therefore expect the projection *π*: *Z*_*k*_ → *P*(*K*^*k*^) to be dominant for *k* ≫ *n*. To prove that this is indeed the case, we need to show that for *z* ∈ *Z*_*k*_ sufficiently general, the local dimension at *z* of the fibre *π*^− 1^(*π*(*z*)) is (at most) $\dim (Z_{k})-e_{k}=kn-e_{n}$. By the upper semicontinuity of the fibre dimension [18, Theorem 11.12], it suffices to exhibit a single point *z* with this property, and indeed, it suffices to show that the tangent space to the fibre at *z* has dimension (at most) *kn* − *e*_*n*_.

To find such a point *z*, set *U* := *K*^*n*^ and *V* := *K*^*k*−*n*^ and consider

$$ z:=(\pi_{U},P(\iota_{U})q + r) \in Z_{k}, $$

where *π*_*U*_: *U* ⊕ *V* → *U* is the projection and *ι*_*U*_: *U* → *U* ⊕ *V* is the inclusion and where we will choose $r \in P^{\prime }(U) \otimes V \subseteq P(U \oplus V)$. Note that then

$$ P(\iota_{U})q + r \in P(U) \oplus (P^{\prime}(U) \otimes V) \subseteq P(U \oplus V) $$

and that *P*(*π*_*U*_)*r* = 0 so that *z* does, indeed, lie in *Z*_*k*_.

The tangent space $T_{z} {\varPi }^{-1}({\varPi }(z))$ (projected into Hom(*K*^*k*^,*K*^*n*^)) is contained in the solution space of the linear system of equations

$$ P(\pi_{U} + \varepsilon \psi)(P(\iota_{U}) q + r) = q \mod \varepsilon^{2} $$

for *ψ*. The dimension of this solution space equals $kn=\dim (\text {Hom}(K^{k},K^{n}))$ minus the rank of the linear map

$$ \text{Hom}(U \oplus V,U) \to P(U),\quad \psi \mapsto \text{ the coefficient of $\varepsilon$ in } P(\pi_{U} + \varepsilon \psi)(P(\iota_{U}) q + r). $$

So it suffices to prove that for all *k* ≫ *n* there is a suitable *r* such that this linear map is surjective. In fact, we will restrict the domain to those *ψ* ∈Hom(*U* ⊕ *V*, *U*) of the form *ω* ∘ *π*_*V*_ where *π*_*V*_: *U* ⊕ *V* → *V* is the projection and *ω* ∈Hom(*V*, *U*). Then

$$ P(\pi_{U} + \varepsilon \psi)(P(\iota_{U}) q) =P((\pi_{U} + \varepsilon \omega\circ \pi_{V})\circ\iota_{U}) q=P(\text{id}_{U})q=q. $$

So *P*(*ι*_*U*_)*q* does not contribute to the coefficient of *ε* and this coefficient equals

$$ P(\text{id}_{U} + \text{id}_{U})\left( \text{id}_{P^{\prime}(U)} \otimes \omega\right) r, $$

where id_*U*_ + id_*U*_: *U* ⊕ *U* → *U* is the map sending (*u*_1_,*u*_2_) to *u*_1_ + *u*_2_. Note that the codomain of $\text {id}_{P^{\prime }(U)} \otimes \omega $ equals $P^{\prime }(U) \otimes U \subseteq P(U \oplus U)$, so that the composition above makes sense. Below we will show that for $k-n = \dim V \gg n$ and suitable $r \in P^{\prime }(U) \otimes V$ the linear map

$$ \begin{array}{@{}rcl@{}} {\varOmega}_{P,V,r} \colon \text{Hom}(V,U) &\to& P(U)\\ \omega &\mapsto& P(\text{id}_{U} + \text{id}_{U})\left( \text{id}_{P^{\prime}(U)} \otimes \omega\right)r \end{array} $$

is surjective.

Hence, there exists a *k* such that *Z*_*k*_ → *P*(*K*^*k*^) is dominant. By Chevalley’s theorem, the image contains a dense open subset of *P*(*K*^*k*^), and this dense open subset intersects the dense set *X*(*K*^*k*^). Hence, there exists an element *p* ∈ *X*(*K*^*k*^) and a *φ* ∈Hom(*K*^*k*^,*K*^*n*^) such that *P*(*ϕ*)*p* = *q*. Finally, since *X* is a subset of *P*, also *q* is a point in *X*(*K*^*n*^). Hence, *X*(*K*^*n*^) = *P*(*K*^*n*^) for each *n*, as desired. □

#### **Lemma 3.2.2**

Let *P* be a polynomial functor over an infinite field *K* with char(*K*) = 0 or char(*K*) > deg(*P*) and let *U* ∈**Vec**. Then for *V* ∈**Vec** with $\dim V \gg \dim U$, there exists an $r \in P^{\prime }(U) \otimes V$ such that

$$ \begin{array}{@{}rcl@{}} {\varOmega}_{P,V,r}\colon\text{Hom}(V,U) &\to& P(U)\\ \omega &\mapsto& P(\text{id}_{U} + \text{id}_{U})\left( \text{id}_{P^{\prime}(U)} \otimes \omega\right)r \end{array} $$

is surjective.

#### *Proof*

When char(*K*) = 0, the Abelian category of polynomial functors is semisimple, with the Schur functors as a basis. When char(*K*) = *p* > 0, the situation is more complicated. The irreducible polynomial functors still correspond to partitions [17, Theorem 3.5]. A degree-*d* irreducible polynomial functor is a submodule of the functor *T*(*V* ) = *V*^⊗*d*^ if and only if the corresponding partition is column *p*-regular [21, Theorem 3.2]. Luckily, this is always the case when *d* < *p*. And, the Abelian category of polynomial functors of degree < *p* is semisimple [17, Corollary 2.6e]. Now, if *P*, *Q* are such polynomial functors and $r_{1} \in P^{\prime }(U) \otimes V$ and $r_{2} \in Q^{\prime }(U) \otimes W$ have the required property for *P*, *Q*, respectively, then

$$ \begin{array}{@{}rcl@{}} r:=(r_{1},r_{2}) \in (P^{\prime}(U) \otimes V) \oplus (Q^{\prime}(U) \otimes W) &\subseteq& (P^{\prime}(U) \oplus Q^{\prime}(U)) \otimes (V \oplus W)\\ & =& (P\oplus Q)'(U) \otimes (V \oplus W) \end{array} $$

has the required property for *P* ⊕ *Q*. Hence, it suffices to prove the lemma in the case where *P* is an irreducible polynomial functor of degree *d*. We then have *T* = *P* ⊕ *Q*, where *T*(*V* ) = *V*^⊗*d*^ and *Q* is another polynomial functor. By a similar argument as above, if $r \in T^{\prime }(U) \otimes V$ has the required property for *T*, then its image in $P^{\prime }(U) \otimes V$ has the required property for *P*. Hence, it suffices to prove the lemma for *T*.

Now we have

$$ \begin{array}{@{}rcl@{}} T(U \oplus V)=T(U) &\oplus& (V \otimes U \otimes U \otimes {\cdots} \otimes U) \oplus (U \otimes V \otimes U \otimes {\cdots} \otimes U) \\ &\oplus& {\cdots} \oplus (U \otimes U \otimes U \otimes {\cdots} \otimes V) \oplus \text{ terms of higher degree in \textit{V},} \end{array} $$

so that $T^{\prime }$ is a direct sum of *d* copies of *U*↦*U*^⊗*d*− 1^. We take *r* in the first of these copies, as follows. Let *e*_1_,…,*e*_*n*_ be a basis of *U* and set

$$ r:=\underset{\alpha \in [n]^{d-1}}{\sum} v_{\alpha} \otimes e_{\alpha_{1}} \otimes {\cdots} \otimes e_{\alpha_{d-1}}, $$

where the *v*_*α*_ are a basis of a space *V* of dimension *n*^*d*− 1^. For every *β* ∈ [*n*]^*d*− 1^ and *i* ∈ [*n*], the linear map *ω* that maps *v*_*β*_ to *e*_*i*_ and all other *v*_*α*_ to zero is a witness to the fact that $e_{i} \otimes e_{\beta _{1}} \otimes {\cdots } \otimes e_{\beta _{d-1}}$ is in the image of *Ω*_*T*, *V*, *r*_. Hence, this linear map is surjective. □

#### **Lemma 3.2.3**

Assume that *K* is algebraically closed of characteristic zero. Let *P*, *Q* be polynomial functors. Assume that *P* is irreducible of degree *d*, *Q* has degree < *d* and let *α*: *Q* → *P* be a polynomial transformation, then there is a uniform bound on the strength of elements of im(*α*_*V*_) that is independent of *V*.

#### *Proof*

Let *R* be the sum of the components of *Q* of strictly positive degree. Any element in im(*α*_*V*_) is also in im(*β*_*V*_) for a polynomial transformation *β*_*V*_: *R* → *P* obtained from *α* by a suitable specialisation. Write *R* = *R*^(1)^ ⊕⋯ ⊕ *R*^(*k*)^, where the *R*^(*i*)^ are Schur functors of degrees 0 < *d*_*i*_ < *d*. The polynomial transformation *β* factors uniquely as the polynomial transformation

$$ \begin{array}{@{}rcl@{}} \delta\colon R^{(1)} \oplus{\cdots} \oplus R^{(k)}~&\to&~F:=\underset{\begin{array}{cc}e_{1},\ldots,e_{k}\geq0\\{\sum}_{i} e_{i} d_{i}=d \end{array}}{\bigoplus} \bigotimes_{i=1}^{k} S^{e_{i}} R^{(i)} \\ (r_{1},\ldots,r_{k})~&\mapsto&~\left( r_{1}^{\otimes e_{1}}\otimes\cdots\otimes r_{k}^{\otimes e_{k}}\right)_{e_{1},\ldots,e_{k}} \end{array} $$

and a *linear* polynomial transformation *γ*: *F* → *P*. As *γ* is linear, we see that str(*γ*_*V*_(*v*)) ≤str(*v*) for all *V* ∈**Vec** and *v* ∈ *F*(*V* ). So it suffices to prove that the elements of the subset im(*δ*), which depends only on *Q* and *d*, have bounded strength. We have

$$ \text{str}\left( r_{1}^{\otimes e_{1}}\otimes\cdots\otimes r_{k}^{\otimes e_{k}}\right)_{e_{1},\ldots,e_{k}} \!\leq\! \underset{\begin{array}{cc}e_{1},\ldots,e_{k}\geq0\\{\sum}_{i} e_{i} d_{i}=d \end{array}}{\sum}\text{str}\left( r_{1}^{\otimes e_{1}}\otimes\cdots\otimes r_{k}^{\otimes e_{k}}\right) \!\leq\! \underset{\begin{array}{cc}e_{1},\ldots,e_{k}\!\geq\!0\\{\sum}_{i} e_{i} d_{i}=d \end{array}}{\sum} 1 $$

as ${\sum }_{i} e_{i}\geq 2$ whenever ${\sum }_{i} e_{i}d_{i}=d$. So this is indeed the case. □

#### *Proof*

*of Theorem 2.5.2 (Main Theorem I)* Let *X* be a subset of a pure polynomial functor *P* over an algebraically closed field *K* of characteristic zero. For each *V* ∈**Vec** define $Y(V):=\overline {X(V)}$. If *Y* is a proper closed subset of *P*, then by [6, Theorem 4.2.5] there exist finitely many polynomial transformations *α*_*i*_: *Q*_*i*_ → *P* with $Q_{i}\lessdot P$ and $Y(V) \subseteq \bigcup _{i} \text {im}(\alpha _{i,V})$ for all *V* ∈**Vec**. Since $X \subseteq Y$, we are done. Otherwise, if *Y* (*V* ) = *P*(*V* ) for all *V*, then Theorem 3.2.1 implies that also *X*(*V* ) = *P*(*V* ) for all *V*. The last statement follows from the previous lemma. □

#### *Proof*

*of Corollary 2.5.4* Let *X* be the subset of *P* consisting of all elements *f* ∈ *P*(*V* ) such that

$$ \begin{array}{@{}rcl@{}} \text{Hom}(V,U) &\to& P(U)\\ \varphi &\mapsto& P(\varphi)f \end{array} $$

is not surjective. By Main Theorem I, it suffices to prove that *X*≠*P*. As before, we claim that in fact *X*(*V* )≠*P*(*V* ) already when $\dim V\geq \deg (P)\cdot \dim P(U)$.

First suppose that *P* is irreducible. Then *P* is a Schur functor. Take *V*_0_ = *K*^*d*^ and $\ell =\dim P(U)$. Then it is known that Hom(*V*_0_,*U*) ⋅ *P*(*V*_0_) spans *P*(*U*). Let *P*(*φ*_1_)*p*_1_,…,*P*(*φ*_*ℓ*_)*p*_*ℓ*_ be a basis of *P*(*U*), let $\iota _{i}\colon V_{0}\to V_{0}^{\oplus \ell }$ and $\pi _{i}\colon V_{0}^{\ell }\to V_{0}$ be the inclusion and projection maps and take

$$ p=P(\iota_{i})p_{1}+\cdots+P(\iota_{\ell})p_{\ell}\in P(V_{0}^{\oplus \ell}). $$

Then *P*(*φ*_*i*_ ∘ *π*_*i*_)(*p*) = *P*(*φ*_*i*_)*p*_*i*_. Hence,

$$ \begin{array}{@{}rcl@{}} \text{Hom}(V_{0}^{\oplus\ell},U) &\to& P(U)\\ \varphi &\mapsto& P(\varphi)p \end{array} $$

is surjective.

Next, suppose that *P* = *Q* ⊕ *R* and that there exist *f* ∈ *Q*(*V* ) and *g* ∈ *R*(*W*) such that

$$ \begin{array}{rlcrl} \text{Hom}(V,U) &~\to~ Q(U)&\qquad\text{and}\qquad&~\text{Hom}(W,U) &~\to~ R(U)\\ \varphi &~\mapsto~ Q(\varphi)f&\qquad&\varphi&~\mapsto~ R(\varphi)g \end{array} $$

are surjective. By induction, we can assume such *f*, *g* exist when $\dim V \geq \text {deg}(P)\cdot \dim Q(U)$ and $\dim W\geq \text {deg}(P)\cdot \dim R(U)$. Now, we see that

$$ \begin{array}{@{}rcl@{}} \text{Hom}(V\oplus W,U) &\to& P(U)\\ \varphi &\mapsto& P(\varphi)(P(\iota_{1})(f)+P(\iota_{2})(g)) \end{array} $$

is surjective. This proves the first part of the corollary. For the second statement, we note that when *P* is irreducible the elements of im(*α*_*i*_) have bounded strength. As the bound depends only on *X* and *X* only depends on $\dim U$, we see that $f \not \in \bigcup _{i=1}^{k} \text {im}(\alpha _{i})$ for all *f* with strength greater than some function of $\dim U$ only. □

## Proof of Main Theorem II

### Construction of the Minimal Class

Let *P* be a homogeneous polynomial functor of degree *d* > 0 over an algebraically closed field *K* of characteristic zero. Decompose

$$ P=P^{(1)} \oplus {\cdots} \oplus P^{(\ell)} $$

into Schur functors. For each *U* ∈**Vec** of dimension ≥ *d* the GL(*U*)-module *P*^(*i*)^(*U*) is irreducible (and in particular nonzero). Let *V* ∈**Vec** be a vector space of dimension *d*. Let *V*^(1,*i*)^ be a copy of *V* for each *i* = 1,…,*ℓ* and choose any nonzero *q*^(1,*i*)^ ∈ *P*^(*i*)^(*V*^(1,*i*)^). We write

$$ q^{(1)}:=q^{(1,1)} + {\cdots} + q^{(1,\ell)} \in P^{(1)}(V^{(1,1)}) \oplus {\cdots} \oplus P^{(\ell)}(V^{(1,\ell)}) \subseteq P(W^{(1)}), $$

where *W*^(1)^ = *V*^(1,1)^ ⊕⋯ ⊕ *V*^(1,*ℓ*)^. We take independent copies *W*^(*j*)^ = *V*^(*j*,1)^ ⊕⋯ ⊕ *V*^(*j*, *ℓ*)^ of *W*^(1)^ and copies *q*^(*j*)^ = *q*^(*j*,1)^ + ⋯ + *q*^(*j*, *ℓ*)^ ∈ *P*(*W*^(*j*)^) of *q*_1_ and set

$$ q:=q^{(1)} + q^{(2)} + {\cdots} \in P_{\infty}, $$

where we concatenate copies of a basis in the *ℓd*-dimensional space *W*^(1)^ to identify *W*^(1)^ ⊕⋯ ⊕ *W*^(*k*)^ with *K*^*kℓd*^.

#### *Example 4.1.1*

Let $P=S^{d} \oplus \bigwedge ^{d}$, so that we may take *V* = *K*^*d*^. We may take $q^{(1,1)}:={x_{1}^{d}} \in S^{d}(V^{(1,1)})$ and $q^{(1,2)}:=x_{d+1} \wedge {\cdots } \wedge x_{2d} \in \bigwedge ^{d}(V^{(1,2)})$, where *x*_1_,…,*x*_*d*_ and *x*_*d*+ 1_,…,*x*_2*d*_ are bases of *V*^(1,1)^ and *V*^(1,2)^, respectively. We then have

$$ q=({x_{1}^{d}} + x_{d+1}\wedge {\cdots} \wedge x_{2d}) + \left( x_{2d+1}^{d} + x_{3d+1}\wedge {\cdots} \wedge x_{4d}\right) + \cdots $$

We will prove, first, that any *q* constructed in this manner has a dense $\text {GL}_{\infty }$-orbit in $P_{\infty }$, and second, that *q* ≼ *p* for all $p \in P_{\infty }$ with a dense $\text {GL}_{\infty }$-orbit.

### Density of the Orbit of *q*

#### **Proposition 4.2.1**

The $\text {GL}_{\infty }$-orbit of *q* is dense in $P_{\infty }$.

#### *Proof*

It suffices to prove that for each *U* ∈**Vec** and each *p* ∈ *P*(*U*) there exists a *k* ≥ 1 and a linear map *φ*: *W*^(1)^ ⊕⋯ ⊕ *W*^(*k*)^ → *U* such that *P*(*φ*)(*q*^(1)^ + ⋯ + *q*^(*k*)^) = *p*. Furthermore, we may assume that *U* has dimension at least *d*. Fix a linear injection *ι*: *V* → *U*. Now $\tilde {q}^{(i)}:=P(\iota )(q^{(j,i)})$ is a nonzero vector in the GL(*U*)-module *P*^(*i*)^(*U*), which is irreducible. Hence, the component *p*^(*i*)^ of *p* in *P*^(*i*)^(*U*) can be written as

$$ p^{(i)}=P\left( g^{(1,i)}\right)\tilde{q}^{(i)} + {\cdots} + P\left( g^{(k_{i},i)}\right)\tilde{q}^{(i)} $$

for suitable elements $g^{(1,i)},\ldots ,g^{(k_{i},i)} \in \text {End}(U)$. Do this for all *i* = 1,…,*ℓ*. By taking the maximum of the numbers *k*_*i*_ (and setting the irrelevant *g*^(*j*, *i*)^ equal to zero) we may assume that the *k*_*i*_ are all equal to a fixed number *k*; this is the *k* that we needed. Now we may define *φ* by declaring its restriction on *V*^(*j*, *i*)^ to be equal to *g*^(*j*, *i*)^ ∘ *ι*. We then have

$$ P(\varphi)(q_{1}+\cdots+q_{k}) = \sum\limits_{j=1}^{k} \sum\limits_{i=1}^{\ell} P\left( g^{(j,i)}\right) \tilde{q}^{(i)} = \sum\limits_{i=1}^{\ell} p^{(i)} = p, $$

as desired. □

### Minimality of the Class of *q*

#### **Proposition 4.3.1**

We have *q* ≼ *p* for every $p \in P_{\infty }$ with a dense $\text {GL}_{\infty }$-orbit.

#### *Proof*

Let $p \in P_{\infty }$ be a tensor with a dense $\text {GL}_{\infty }$-orbit and write *p* = (*p*_0_,*p*_1_,*p*_2_,…) with *p*_*i*_ ∈ *P*(*K*^*i*^). Take *m*_0_ = *n*_0_ = 0. There exists a linear map $\varphi _{0}\colon K^{m_{0}}\to K^{n_{0}}$ such that $P(\varphi _{0})p_{m_{0}}=q_{n_{0}}=0$, namely the zero map. Write *n*_*i*_ = *n*_0_ + *iℓd*. Our goal is to construct, for each integer *i* ≥ 1, an integer *m*_*i*_ ≥ *m*_*i*− 1_ and a linear map $\psi _{i}\colon K^{[m_{i}]-[m_{i-1}]}\to W^{(i)}$ such that the linear map $\varphi _{i}\colon K^{m_{i}}\to K^{n_{i}}$ making the diagram 
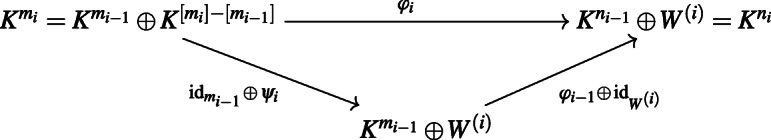


commute satisfies $P(\varphi _{i})p_{m_{i}}=q_{n_{i}}=q^{(1)}+\cdots +q^{(i)}$.

Let *i* ≥ 1 be an integer. As observed in Section 3.1, we can write

$$ P(K^{m_{i-1}} \oplus V) = P(K^{m_{i-1}})\oplus R_{1}(V)\oplus\cdots\oplus R_{d-1}(V)\oplus P(V), $$

where $R_{j}=\text {Sh}_{K^{m_{i-1}}}(P)_{j}$ is a homogeneous polynomial functor of degree *j*. Writing $K^{\mathbb {N}}$ as $K^{m_{i-1}} \oplus K^{\mathbb {N}-[m_{i-1}]}$, we obtain a corresponding decomposition

$$ p=p_{m_{i-1}} + r_{1} + {\cdots} + r_{d-1} + p^{\prime}, $$

where $r_{j}\in R_{j,\infty -m_{i-1}}$ and $p^{\prime }\in P_{\infty -m_{i-1}}$ and we claim that $p^{\prime }$ has a dense $\text {GL}_{\infty -m_{i-1}}$-orbit; here we use the notation from Remark 2.6.7.

The polynomial bifunctor (*U*, *V* )↦*P*(*U* ⊕ *V* ) is a direct sum of bifunctors of the form (*U*, *V* )↦*Q*(*U*) ⊗ *R*(*V* ) where *Q*, *R* are Schur functors. It follows that *R*_*j*_(*V* ) is the direct sum of spaces $Q(K^{m_{i-1}})\otimes R(V)$ where *Q*, *R* are Schur functors of degrees *d* − *j*, *j*, respectively. Hence the elements *r*_1_,…,*r*_*d*− 1_ have finite strength. Also note that $p_{m_{i-1}}\in P(K^{m_{i-1}})$ has finite strength. So by Corollary 2.6.8, we see that the $\text {GL}_{\infty -m_{i-1}}$-orbit of $p^{\prime }$ must be dense.

The tuple $(r_{1},\ldots ,r_{d-1}) \in \bigoplus _{j=1}^{d-1}R_{j,\infty -m_{i-1}}$ may not have a dense $\text {GL}_{\infty -m_{i-1}}$-orbit. However, there exists a polynomial functor *R* less than or equal to *R*_1_ ⊕⋯ ⊕ *R*_*d*− 1_ with *R*({0}) = {0}, an $r \in R_{\infty -m_{i-1}}$ and a polynomial transformation

$$ \alpha=(\alpha_{1},\ldots,\alpha_{d-1})\colon R \to R_{1} \oplus {\cdots} \oplus R_{d-1} $$

such that *r* has a dense $\text {GL}_{\infty -m_{i-1}}$-orbit and *α*(*r*) = (*r*_1_,…,*r*_*d*− 1_). Since *P* is homogeneous of degree *d* > deg(*R*), the pair $(r,p^{\prime })$ has a dense orbit in $R_{\infty -m_{i-1}} \oplus P_{\infty -m_{i-1}}$ by [6, Lemma 4.5.3]. Hence, by Corollary 2.6.3, there exists an *m*_*i*_ ≥ *m*_*i*− 1_ + *ℓd* and a linear map $\psi _{i}\colon K^{[m_{i}]-[m_{i-1}]} \to W^{(i)}$ such that $R(\psi _{i})r_{[m_{i}]-[m_{i-1}]} = 0$ and $P(\psi _{i})p^{\prime }_{[m_{i}]-[m_{i-1}]}=q^{(i)}$.

Since polynomial transformations between polynomial functors with zero constant term map zero to zero, the first equality implies that, for all *j* = 1,…,*d* − 1,

$$ R_{j}(\psi_{i})r_{j,[m_{i}]-[m_{i-1}]}=R_{j}(\psi_{i})\alpha_{j}\left( r_{[m_{i}]-[m_{i-1}]}\right) = \alpha_{j}\left( R(\psi_{i})r_{[m_{i}]-[m_{i-1}]}\right) = \alpha_{j}(0)=0. $$

Thus, informally, applying the map *ψ*_*i*_ makes $p^{\prime }$ specialise to the required *q*^(*i*)^, while the terms *r*_1_,…,*r*_*d*− 1_ are specialised to zero.

We define *φ*_*i*_ as above and we have

$$ \begin{array}{@{}rcl@{}} P(\varphi_{i})p_{m_{i}}& = &P\left( \varphi_{i-1}\oplus\text{id}_{W^{(i)}}\right) P\left( \text{id}_{m_{i-1}}\oplus \psi_{i}\right)\left( p_{m_{i-1}} + \sum\limits_{j=1}^{d-1}r_{j,[m_{i}]-[m_{i-1}]} + p^{\prime}_{[m_{i}]-[m_{i-1}]}\right)\\ & = &P\left( \varphi_{i-1}\oplus\text{id}_{W^{(i)}}\right) \left( p_{m_{i-1}} + \sum\limits_{j=1}^{d-1}R_{j}(\psi_{i})r_{j,[m_{i}]-[m_{i-1}]}+ P(\varphi_{i})p^{\prime}_{[m_{i}]-[m_{i-1}]}\right)\\ & = &P\left( \varphi_{i-1}\oplus\text{id}_{W^{(i)}}\right)\left( p_{m_{i-1}}+q^{(i)}\right) = q_{n_{i-1}}+q^{(i)}=q^{(1)}+\cdots+q^{(i)}. \end{array} $$

Iterating this argument, we find that the infinite matrix

$$ \begin{pmatrix}\varphi_{0}\\&\psi_{1}\\&&\psi_{2}\\&&&\psi_{3}\\&&&&\ddots\end{pmatrix}=: e $$

has the property that *P*(*e*)*p* = *q*^(1)^ + *q*^(2)^ + ⋯ = *q*, as desired. □

#### *Remark 4.3.2*

Note that the element *e* ∈ *E* constructed above has only finitely many nonzero entries in each row *and* in each column!

#### *Remark 4.3.3*

Fix an integer *k* ≥ 0. Then we have the following strengthening of the previous theorem: we have (*x*_1_,…,*x*_*k*_,*q*) ≼ (*ℓ*_1_,…,*ℓ*_*k*_,*p*) for every $(\ell _{1},\ldots ,\ell _{k},p)\in (S^{1}_{\infty })^{\oplus k}\oplus P_{\infty }$ with a dense $\text {GL}_{\infty }$-orbit. Here *q* is defined as before in variables distinct from *x*_1_,…,*x*_*k*_. To see this, note that a tensor in $(S^{1}_{\infty })^{\oplus k}\oplus P_{\infty }$ with a dense $\text {GL}_{\infty }$-orbit is of the form (*ℓ*_1_,…,*ℓ*_*k*_,*p*) where $\ell _{1},\ldots ,\ell _{k}\in S^{1}_{\infty }$ are linearly independent and $p\in P_{\infty }$ has a dense $\text {GL}_{\infty }$-orbit. By acting with an invertible element of *E* as in Example 2.8.2, we may assume that *ℓ*_*i*_ = *x*_*i*_. Take *n*_0_ = *k*. Similar to induction step in the proof of the previous theorem, there exists an integer *m*_0_ ≥ *k* and a linear map $\psi \colon K^{[m_{0}]-[k]}\to K^{n_{0}}$ such that the linear map $\varphi _{0}=\text {id}_{k}+\psi \colon K^{k}\oplus K^{[m_{0}]-[k]}\to K^{n_{0}}$ satisfies $P(\varphi _{0})p_{m_{0}}=q_{n_{0}}=0$. We now proceed as in the proof of the theorem with these *m*_0_,*n*_0_,*φ*_0_ to find the result.

#### *Proof*

*of Theorem 2.9.1, existence of* *p*. The existence of a minimal *p* among all elements with a dense $\text {GL}_{\infty }$-orbit follows directly from Propositions 4.2.1 and 4.3.1. □

### Maximal Tensors

Next, we construct maximal elements with respect to ≼ of $P_{\infty }$ for any pure polynomial functor *P*. We start with *n*-way tensors, then do Schur functors and finally general polynomial functors. Let *d* ≥ 1 be an integer and let *T*^*d*^ be the polynomial functor sending *V* ↦*V*^⊗*d*^.

#### **Lemma 4.4.1**

There exists a tensor $r_{d}\in T^{d}_{\infty }$ such that *p* ≼ *r*_*d*_ for all $p\in T^{d}_{\infty }$.

#### *Proof*

For *d* = 1, we know that the element $r_{1}:=x_{1}\in T^{1}_{\infty }$ satisfies *p* ≼ *r*_1_ for all $p\in T^{1}_{\infty }$. Now suppose that *d* ≥ 2 and that $r_{d-1}=r_{d-1}(x_{1},x_{2},\ldots )\in T^{d-1}_{\infty }$ satisfies *p* ≼ *r*_*d*− 1_ for all $p\in T^{d-1}_{\infty }$. We define a $r_{d}\in T^{d}_{\infty }$ satisfying *p* ≼ *r*_*d*_ for all $p\in T^{d}_{\infty }$.

For *j* ∈{1,…,*d*}, we define the map $-\otimes _{j}-\colon T^{1}_{\infty }\times T^{d-1}_{\infty }\to T^{d}_{\infty }$ as the inverse limit of the bilinear maps −⊗_*j*_−: *V* × *V*^⊗*d*− 1^ → *V*^⊗*d*^ such that

$$ v_{j}\otimes_{j}(v_{1}\otimes {\cdots} \otimes v_{j-1}\otimes v_{j+1}\otimes\cdots\otimes v_{d})=v_{1}\otimes\cdots\otimes v_{d} $$

for all finite-dimensional vector space *V* and all vectors *v*_1_,…,*v*_*d*_ ∈ *V*. Now, we take

$$ r_{d}:=\sum\limits_{i=1}^{\infty}\sum\limits_{j=1}^{d}x_{\iota(i,j,1)}\otimes_{j} r_{d-1}\left( x_{\iota(i,j,2)},x_{\iota(i,j,3)},\ldots\right), $$

where $\iota \colon \mathbb {N}\times \{1,\ldots ,d\}\times \mathbb {N}\to \mathbb {N}$ is any injective map. We claim that *p* ≼ *r*_*d*_ for all $p\in T^{d}_{\infty }$. Indeed, any such *p* can we written as

$$ p=\sum\limits_{i=1}^{\infty}\sum\limits_{j=1}^{d}x_{i}\otimes_{j} p_{i}(x_{i},x_{i+1},\ldots) $$

with $p_{1},p_{2},\ldots \in T^{d-1}_{\infty }$ and by assumption we can specialise *r*_*d*− 1_ to *p*_*i*_ using an element of *E* for all *i*. Combined, this yields a specialisation of *r*_*d*_ to *p*. Note here that *x*_*ι*(*i*, *j*,1)_↦*x*_*i*_ and *x*_*ι*(*i*, *j*, *k*)_↦*ℓ*_*i*, *j*, *k*_ for *k* > 1 in such a way that *x*_*ℓ*_ occurs, when ranging over *k*, in only finitely many *ℓ*_*i*, *j*, *k*_ when *i* ≤ *ℓ* and *x*_*ℓ*_ does not occur in *ℓ*_*i*, *j*, *k*_ when *i* > *ℓ*. This means that the specialisation of *r*_*d*_ to *p* indeed goes via an element of *E*. So for all *d* ≥ 1, the space $T^{d}_{\infty }$ has a maximal element with respect to ≼. □

#### **Lemma 4.4.2**

Let *P* be a Schur functor of degree *d* ≥ 1. Then there exists a tensor $r\in P_{\infty }$ such that *p* ≼ *r* for all $p\in P_{\infty }$.

#### *Proof*

The space $P_{\infty }$ is a direct summand of $T^{d}_{\infty }$. Let *r* be the component in $P_{\infty }$ of *r*_*d*_ from the previous lemma. Then *p* ≼ *r* for all $p\in P_{\infty }$. □

#### **Proposition 4.4.3**

Let *P* be a pure polynomial functor. Then there exists a tensor $r\in P_{\infty }$ such that *p* ≼ *r* for all $p\in P_{\infty }$.

#### *Proof*

Write

$$ P=P^{(1)}\oplus\cdots\oplus P^{(k)} $$

as a direct sum of Schur functors. For each *i* ∈{1,…,*k*}, let $r_{i} = r_{i}(x_{1},x_{2},\ldots )\in P^{(i)}_{\infty }$ be a tensor such that *p*_*i*_ ≼ *r*_*i*_ for all $p_{i}\in P^{(i)}_{\infty }$ and take $r=(r_{1}(x_{1},x_{k+1},\ldots ),\ldots ,r_{k}(x_{k},x_{2k},\ldots ))\in P_{\infty }$. Then *p* ≼ *r* for all $p\in P_{\infty }$. □

#### *Proof*

*of Theorem 2.9.1, the existence of* *r*. This follows directly from Proposition 4.4.3. □

## Further Examples

In this section we give more examples: we prove that tensors in $P_{\infty }$ with a dense $\text {GL}_{\infty }$-orbit for a single equivalence class when *P* has degree ≤ 2, we compare candidates for minimal tensors in a direct sum of *S*^*d*^’s of distinct degrees and we construct maximal elements in $P_{\infty }$ for all *P* with *P*({0}) = {0}.

### Polynomial Functors of Degree ≤ 2

#### *Example 5.1.1*

Take *P* = *S*^1^ ⊕ *S*^1^. Then a pair $(v,w)\in S^{1}_{\infty }\oplus S^{1}_{\infty }$ has one of the following forms:

1. the pair (*v*, *w*) with $v,w\in S^{1}_{\infty }$ linearly independent vectors;
2. the pair (*λu*, *μu*) with $u\in S^{1}_{\infty }$ nonzero and $[\lambda :\mu ]\in \mathbb {P}^{1}$; or
3. the pair (0,0).

In the first case, the pair (*v*, *w*) has a dense $\text {GL}_{\infty }$-orbit and is equivalent to (*x*_1_,*x*_2_). When *μv* − *λw* = 0 for some *λ*, *μ* ∈ *K*, then this also holds for all specialisations of (*v*, *w*). So the poset of equivalence classes is given by: 
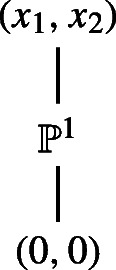


where a point $[\lambda :\mu ]\in \mathbb {P}^{1}$ corresponds to the class of (*λu*, *μu*) with $u \in S^{1}_{\infty }$ nonzero and all points in $\mathbb {P}^{1}$ are incomparable.

#### *Example 5.1.2*

Take *P* = *S*^2^. By Proposition 4.3.1 each infinite quadric

$$ p={\sum}_{1 \leq i \leq j} a_{ij} x_{i} x_{j} $$

of infinite rank specialises to the quadric *q* = *x*_1_*x*_2_ + *x*_3_*x*_4_ + ⋯ via a suitable linear change of coordinates. Here each variable is only allowed to occur in only finitely many of the linear forms that *x*_1_,*x*_2_,… are substituted by. Conversely, it is not difficult to see that *q* specialises to *p* as well by applying the following element of *E*:

$$ \left( \begin{array}{ccccccc} 1 & a_{11} & 0 & 0  & 0 & 0 & \cdots\\ 0 & a_{12} & 1 & a_{22} & 0 & 0 & \cdots\\ 0 & a_{13} & 0 & a_{23} & 1 & a_{33} &\cdots\\ 0 & a_{14} & 0 & a_{24} & 0 & a_{34} &\cdots\\ {\vdots} & {\vdots} & {\vdots} & {\vdots} & {\vdots} & {\vdots} & \end{array}\right). $$

We conclude that the infinite-rank quadrics form a single equivalence class under $\simeq $ and that the rank function is an isomorphism from the poset of equivalence classes to the well-ordered set $\{0,1,2,\ldots ,\infty \}$.

#### *Example 5.1.3*

Take $P=\bigwedge ^{2}$. By Proposition 4.3.1 each infinite alternating tensor

$$ p=\underset{1 \leq i < j}{\sum} a_{ij} x_{i}\wedge x_{j} $$

of infinite rank specialises to *q* = *x*_1_ ∧ *x*_2_ + *x*_3_ ∧ *x*_4_ + ⋯. And, *q* specialises to *p* as well by applying the following element of *E*:

$$ \left( \begin{array}{ccccccc} 1 & 0 & 0 & 0  & 0 & 0 & \cdots\\ 0 & a_{12} & 1 & 0 & 0 & 0 & \cdots\\ 0 & a_{13} & 0 & a_{23} & 1 & 0 &\cdots\\ 0 & a_{14} & 0 & a_{24} & 0 & a_{34} &\cdots\\ {\vdots} & {\vdots} & {\vdots} & {\vdots} & {\vdots} & {\vdots} & \end{array}\right). $$

As before, we conclude that the infinite-rank alternating tensors form a single $\simeq $-equivalence class and that the rank function is an isomorphism from the poset of equivalence classes to the well-ordered set $\{0,1,2,\ldots ,\infty \}$.

#### *Example 5.1.4*

Take $P=(S^{1})^{\oplus a}\oplus (S^{2})^{\oplus b}\oplus (\bigwedge ^{2})^{\oplus c}$ for integers *a*, *b*, *c* ≥ 0. By Remark 4.3.3, any tuple in $P_{\infty }$ with a dense $\text {GL}_{\infty }$-orbit specialises to the tuple

$$ \begin{array}{@{}rcl@{}} &&(x_{1},\ldots,x_{a},y_{1}y_{2}+y_{2b+1}y_{2b+2}+\cdots,\ldots,y_{2b-1}y_{2b}+y_{4b-1}y_{4b}+\cdots,\\ &&\qquad\qquad z_{1}\wedge z_{2}+z_{2c+1} \wedge z_{2c+2}+\cdots,\ldots,z_{2c-1}\wedge z_{2c}+z_{4c-1}\wedge z_{4c}+\cdots), \end{array} $$

where *y*_2*ib*+*j*_ = *x*_*a*+ 2*ib*+ 2*ic*+*j*_ for *i* ≥ 0 and 1 ≤ *j* < 2*b* and *z*_2*ic*+*j*_ = *x*_*a*+ 2(*i*+ 1)*b*+ 2*ic*+*j*_ for *i* ≥ 0 and 1 ≤ *j* < 2*c*. By the previous examples, each of the entries in this latter tuple independently specialises to any tensor in the same space. So the entire tuple also specialises to any other tuple in $P_{\infty }$. So the tuple with a dense $\text {GL}_{\infty }$-orbit again form a single $\simeq $-equivalence class.

### Non-homogeneous Polynomial Functors

The proof of Proposition 4.3.1 relies on the fact that *P* is homogeneous. Apart from the slight generalisation from Remark 4.3.3, we do not know if such a result holds in a more general setting.

#### *Question 5.2.1*

Take *P* = *S*^2^ ⊕ *S*^3^. Does there exist a tensor $q\in P_{\infty }$ with a dense $\text {GL}_{\infty }$-orbit such that *q* ≼ *p* for all $p\in P_{\infty }$ with a dense $\text {GL}_{\infty }$-orbit?

The next example compares different candidates for such a minimal element.

#### *Example 5.2.2*

Take $P = S^{d_{1}} \oplus S^{d_{2}} \oplus {\cdots } \oplus S^{d_{k}}$ with 1 < *d*_1_ < ⋯ < *d*_*k*_. By [6, Lemma 4.5.3], an element $(f_{1}, \dots , f_{k})\in P_{\infty }$ has dense $\text {GL}_{\infty }$-orbit if and only if $f_{i} \in S^{d_{i}}_{\infty }$ has dense $\text {GL}_{\infty }$-orbit for all *i* = 1,…,*k*. In particular, the elements

$$ q =\left( q^{(1)},\ldots,q^{(k)}\right) = \left( x_{1}^{d_{1}} + x_{2}^{d_{1}} + \cdots, \ldots, x_{1}^{d_{k}} + x_{2}^{d_{k}} + \cdots\right) $$

and

$$ p=\left( p^{(1)},\ldots,p^{(k)}\right)= \left( x_{1}^{d_{1}} + x_{k+1}^{d_{1}} + \cdots, \dots, x_{k}^{d_{k}} + x_{2k}^{d_{k}} + \cdots\right) $$

have dense $\text {GL}_{\infty }$-orbits. Clearly *q* ≼ *p*. By Corollary 2.6.3, there exists an *n* ≥ 1 and linear forms *ℓ*_1_,…,*ℓ*_*n*_ in *x*_1_,…,*x*_*k*_ such that $q_{n}^{(j)}(\ell _{1},\ldots ,\ell _{n})= x_{j}^{d_{j}}$ for *j* = 1,…,*k*. Take

$$ \ell_{hn+i}=\ell_{i}(x_{hn+1},\ldots,x_{hn+n}) $$

for *h* ≥ 1 and *i* ∈{1,…,*k*}. Then we see that $q_{n}^{(j)}(\ell _{hn+1},\ldots ,\ell _{hn+n})= x_{hn+j}^{d_{j}}$ for *j* = 1,…,*k*. So since

$$ q^{(j)}=q_{n}^{(j)}+q_{n}^{(j)}(x_{n+1},\ldots,x_{2n})+\cdots $$

we see that *q*^(*j*)^(*ℓ*_1_,*ℓ*_2_,…) = *p*^(*j*)^. Let *A* be the *k* × *n* matrix corresponding to *ℓ*_1_,…,*ℓ*_*n*_ and take

$$ e:=\left( \begin{array}{ccc}A\\&A\\&&\ddots \end{array}\right)\in E. $$

Then *P*(*e*)*q*^(*j*)^ = *q*^(*j*)^(*ℓ*_1_,*ℓ*_2_,…). So *p* ≼ *q*. Hence $p\simeq q$.
